# Mechanism of resistance to phagocytosis and pulmonary persistence in mucoid *Pseudomonas aeruginosa*

**DOI:** 10.3389/fcimb.2023.1125901

**Published:** 2023-03-15

**Authors:** Warren J. Rowe, Deborah A. Lebman, Dennis E. Ohman

**Affiliations:** ^1^ Department of Microbiology & Immunology, Virginia Commonwealth University Medical Center, Richmond, VA, United States; ^2^ Research Service, McGuire Veterans Affairs Medical Center, Richmond, VA, United States

**Keywords:** exopolysaccharide, biofilm, mucoid, alginate, phagocytic evasion

## Abstract

**Introduction:**

Pseudomonas aeruginosa is known for its ability to form biofilms, which are dependent on the production of exopolysaccharides. During chronic colonization of the airway and biofilm formation, P. aeruginosa converts to a mucoid phenotype, indicating production of the exopolysaccharide alginate. The mucoid phenotype promotes resistance to phagocytic killing, but the mechanism has not been established.

**Methods and Results:**

To better understand the mechanism of phagocytic evasion conferred by alginate production, Human (THP-1) and murine (MH-S) macrophage cell lines were used to determine the effects of alginate production on macrophage binding, signaling and phagocytosis. Phagocytosis assays using mucoid clinical isolate FRD1 and its non-mucoid algD mutant showed that alginate production inhibited opsonic and non-opsonic phagocytosis, but exogenous alginate was not protective. Alginate caused a decrease in binding to murine macrophages. Blocking antibodies to CD11b and CD14 showed that these receptors were important for phagocytosis and were blocked by alginate. Furthermore, alginate production decreased the activation of signaling pathways required for phagocytosis. Mucoid and non-mucoid bacteria induced similar levels of MIP-2 from murine macrophages.

**Discussion:**

This study demonstrated for the first time that alginate on the bacterial surface inhibits receptor-ligand interactions important for phagocytosis. Our data suggest that there is a selection for alginate conversion that blocks the earliest steps in phagocytosis, leading to persistence during chronic pulmonary infections.

## Introduction

*Pseudomonas aeruginosa* is a gram-negative, biofilm-forming, aerobic rod that is commonly found throughout the environment in soil, surface water, sewage, plants, and foods. It is also an opportunistic pathogen capable of causing disease in multiple tissues including eye, skin, urinary tract, upper and lower respiratory tracts, and blood. Immunocompromised patients, including those on mechanical ventilation, are at risk for serious infections by *P. aeruginosa* (Sadikot et al., 2005). Hospital acquired infections are common, and patients with chronic obstructive pulmonary disease (COPD) are at risk for chronic pulmonary infection (Murphy, 2009). In patients with cystic fibrosis (CF), chronic *P. aeruginosa* infection causes significant inflammation and tissue damage and thus is a major cause of morbidity and mortality (Sadikot et al., 2005).

CF is a major respiratory disease that affects 70,000 people worldwide with 1,000 people being diagnosed every year. It is an autosomal recessive disease caused by a mutation in the cystic fibrosis transmembrane conductance regulator (CFTR) gene, which encodes a chloride ion channel (Folkesson et al., 2012). The loss of this channel results in defective chloride ion transport, leading to the production of a highly adhesive mucus, which inhibits bacterial clearance and promotes respiratory infections (Rubin, 2007). One study found that 97% of children with CF were colonized with *P. aeruginosa* by the age of 3 years; among all CF patients, over 58% are chronically infected with *P. aeruginosa* (Sadikot et al., 2005). *P. aeruginosa* infection in CF is associated with increased morbidity and mortality (Sadikot et al., 2005). Due to the widespread presence of *P. aeruginosa* in the environment and the high incidence of hospital acquired infections, it is important to understand the survival mechanisms responsible for its persistence within the CF lung (Sadikot et al., 2005).

*P. aeruginosa* strains isolated from patients with CF and COPD are often phenotypically different from those isolated from the environment, most prominent of which is their mucoid colony morphology. During colonization of the airway, *P. aeruginosa* undergoes mucoid conversion whereby a normally nonmucoid strain changes to a highly mucoid phenotype, which is indicative of the over production of a capsular exopolysaccharide called alginate (Govan and Deretic, 1996). Alginate is high molecular weight polymer of D-mannuronic acid and L-guluronic acid, where the D-mannuronate residues are also acetylated. Alginate is synthesized and secreted *via* a 12 gene cluster known as the *algD* operon. High levels of alginate are found in the sputum of patients with CF who are chronically infected with *P. aeruginosa* (Folkesson et al., 2012). Also, CF strains often lack flagellin and pilin, and produce ‘rough’ lipopolysaccharides (LPS) that do not have the long O-side chains characteristic of ‘smooth’ LPS (Sadikot et al., 2005). These adaptations promote persistence in the lung apparently by allowing *P. aeruginosa* to evade the host immune system. *P. aeruginosa* is also known for its ability to form biofilms, which are associated with the production of exopolysaccharides.

Alginate production may alter the innate immune response to the infecting organisms. Although alginate production may not be the initial event allowing for persistence or evasion of the immune response, it is likely to be a primary reason for maintaining a chronic infection by inhibiting its clearance by alveolar macrophages. It has been reported that mucoid *P. aeruginosa* is resistant to opsonic and non-opsonic phagocytosis, and that alginate production confers protection against IFN-γ mediated opsonic killing of a *P. aeruginosa* biofilm (Ruhen et al., 1980; Cabral et al., 1987; Krieg et al., 1988; Pier et al., 2001; Leid et al., 2005). Phagocytosis is an actin-dependent process that involves the use of multiple cell surface receptors to bind pathogens (Flannagan et al., 2012; Underhill and Goodridge, 2012). Subsequent internalization requires a complicated series of membrane remodeling, cytoskeletal rearrangements and intracellular signaling events, which also result in the release of inflammatory cytokines.

The mechanism responsible for alginate inhibition of phagocytosis and its effects on subsequent production of inflammatory mediators is not understood. To address how alginate interferes with phagocytosis and its potential effects on subsequent production of inflammatory mediators, we use cell-based host-pathogen interaction assays to compare a mucoid CF-adapted isolate of *P. aeruginosa* to its isogenic nonmucoid mutant, defective in expression of the *algD* operon for alginate biosynthesis. These studies identified macrophage surface receptors that are blocked from ligand binding by alginate expression. Interestingly, inhibition of receptor-ligand interactions and activation of signaling did not affect production of the chemokines MIP-2 and IL-8. Taken together these findings provide additional insight into the mechanisms by which mucoid conversion in *P. aeruginosa* promotes chronic pulmonary disease.

## Results

### Alginate production by Pseudomonas aeruginosa inhibits phagocytosis by human and murine macrophages

Although several studies have shown that mucoid *P. aeruginosa* is more protected from non-opsonoic phagocytosis than nonmucoid strains, the mechanism by which alginate production (Alg^+^) leads to resistance to phagocytosis has not been determined. Thus, to establish the role of mucoid conversion and alginate, the phagocytosis efficiency of isogenic Alg^+/-^ strains of *P. aeruginosa* were compared. FRD1 is a frequently studied CF clinical isolate in which alginate production is constitutively active due to a *mucA* mutation that inactivates the anti-sigma factor of alternative sigma factor, σ^22^. FRD1131 is a mutant of FRD1 that contains a Tn*501-33* insertion mapping near *algD* (Chitnis and Ohman, 1993). The exact site of this insertion was determined in this study and shown to be 16 bp downstream of the start of transcription of the *algD* operon for alginate biosynthesis (data not shown). Thus, Tn*501-33* is polar on all downstream genes, making FRD1131 unable to produce alginate. The Alg^+^ phenotype of FRD1131 was restored by complementation (FRD1131C’) with a plasmid (pALG2) containing the entire *algD* operon. Also, strains were engineered to express green fluorescent protein (GFP) to allow visualization of phagocytosis by fluorescent microscopy and measurement by flow cytometry. A time-course using flow cytometry demonstrated that phagocytosis occurred rapidly, between 15 and 30 min after infection (**Supplemental Figure S1A**). Studies using fluorescent microscopy confirmed that Alg- FRD1131 was taken up much more efficiently than the Alg^+^ parental strain, FRD1 (**Supplemental Figure S1B**).

**Figure 1:**
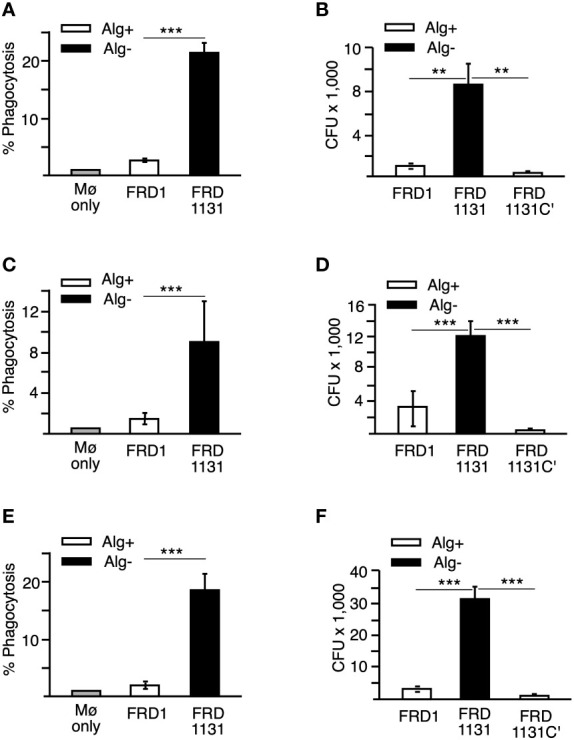
Effects of alginate production on the phagocytosis of *P. aeruginosa*. **(A)** Percentage of THP-1 macrophages that have phagocytized FRD1 and FRD1131 at 30 min. THP-1 macrophages were infected with GFP-expressing Alg^+^ FRD1 or Alg^-^ FRD1131 for 30 min. Percent phagocytosis indicates the percent of macrophages that are associated with GFP-expressing *P. aeruginosa* as determined by flow cytometry. Phagocytosis of FRD1 was significantly inhibited compared to FRD1131. Data are shown as mean and standard deviation of 3 experiments. **(B)** Number of bacteria phagocytized by THP-1 macrophages in 1 h. THP-1 macrophages were infected with FRD1, FRD1131 or FRD1131C’ (Alg^+^) for 1 h. CFU indicates the number of colony-forming units recovered from macrophages. Phagocytosis of FRD1 and FRD1131C’ was significantly inhibited compared to FRD1131. Data are shown as mean and standard deviation of a representative triplicate experiment. **(C)** Percentage of MH-S macrophages that have phagocytized bacteria at 30 min. MH-S macrophages were infected with GFP-expressing FRD1 or FRD1131 for 30 min. Percent phagocytosis indicates the percent of macrophages that are associated with GFP-expressing bacteria as detected by flow cytometry. Phagocytosis of FRD1 was significantly inhibited compared to FRD1131. Data are shown as mean and standard deviation of 3 separate experiments. **(D)** Number of bacteria phagocytized by MH-S macrophages after 1 h. MH-S macrophages were infected with FRD1, FRD1131 or FRD1131C’ for 1 h. CFU indicates the number of colony-forming units recovered from macrophages. Phagocytosis of Alg^+^ FRD1 and FRD1131C’ were significantly inhibited compared to Alg^-^ FRD1131. Data are shown as mean and standard deviation of 3 separate experiments. **(E)** Percentage of MH-S macrophages that have phagocytized opsonized bacteria after 30 min. MH-S macrophages were infected with GFP-expressing FRD or FRD1131 for 30 min in the presence of 0.5% human serum. Percent phagocytosis indicates the percent of macrophages that are associated with GFP-expressing bacteria as detected by flow cytometry. Opsonic phagocytosis of FRD1 was significantly inhibited compared to FRD1131. Data are shown as mean and standard deviation of 3 separate experiments. **(F)** Number of bacteria phagocytized by THP-1 macrophages after 1 hour in the presence of 0.5% human serum. THP-1 macrophages were infected with opsonized FRD1, FRD1131 or FRD1131C’ for 1 h in the presence of 0.5% human serum. CFU indicates the number of colony-forming units recovered from macrophages. Opsonic phagocytosis of Alg^+^ FRD1 and FRD1131C’ were significantly inhibited compared to Alg^-^ FRD1131. Data are shown as mean and standard deviation of a representative triplicate experiment. Statistical significance was determined by ANOVA (* P < 0.05, ** P < 0.01, *** P < 0.001).

Flow cytometry was used to quantitate the effect of alginate production and demonstrated that phagocytosis of Alg^+^ FRD1 was approximately 7-fold reduced compared to Alg^-^ FRD1131 (**Figure 1A**) indicating that alginate plays a major role in resistance to non-opsonic phagocytosis. Since flow cytometry does not distinguish between internalized and bound *P. aeruginosa*, the results were confirmed using the gentamycin protection phagocytosis assay. Here, FRD1 or FRD1131 were incubated with THP-1 macrophages for 1 h, after which macrophages were washed, treated with gentamicin to kill extracellular bacteria, lysed, and intracellular bacteria were quantified by plate counts. The gentamycin protection assay measures the number of bacteria phagocytized, whereas the flow cytometric assay measures the number of macrophages that have phagocytized bacteria. As in the previous assay, phagocytosis of Alg^+^ FRD1 was significantly reduced compared to Alg^-^ FRD1131 (**Figure 1B**). When alginate production was restored in FRD1131 by genetic complementation (FRD1131C’), phagocytosis of FRD1131C’ and FRD1 were similar, suggesting that alginate alone was responsible for the observed resistance to phagocytosis (**Figure 1B**). Together these data indicate that alginate production by *P. aeruginosa* significantly reduced the proportion of macrophages that phagocytized *P. aeruginosa*, as well as the total number of bacteria phagocytized by the macrophages.

During pulmonary disease, *P. aeruginosa* encounters alveolar macrophages, which differ from peripheral macrophages in several important ways including receptor expression, altered activation and survival (Monick et al., 2004; Gwinn and Vallyathan, 2006). Therefore, it was important to determine if alginate production also affected phagocytosis by alveolar macrophages. To investigate this, the murine alveolar macrophage cell line MH-S was used. Similar to the results with THP-1 macrophages, phagocytosis of Alg^+^ FRD1 by MH-S was significantly reduced compared to Alg^-^ FRD1131 with respect to both the percentage of macrophages that phagocytized bacteria (**Figure 1C**) and in the number of bacteria phagocytized (**Figure 1D**). Additionally, Alg^+^ FRD1131C’ was phagocytized significantly less than Alg^-^ FRD1131, suggesting that alginate production alone is responsible for the inhibition of phagocytosis that was observed (**Figure 1D**).

Previous studies investigated the role of alginate in protecting planktonic *P. aeruginosa*, as well as *P. aeruginosa* in a biofilm, from opsonic killing (Ruhen et al., 1980; Cabral et al., 1987; Krieg et al., 1988; Pier et al., 2001; Leid et al., 2005). Since these studies addressed bacterial survival rather than phagocytosis directly, it was of interest to ascertain if alginate also inhibited opsonic phagocytic uptake of planktonic bacteria. Initial studies indicated that at all concentrations of serum (0.5%-10%) FRD1131 was phagocytized to a greater extent than FRD1 (**Supplemental Figure S2**). Like non-opsonic phagoctyosis, opsonic phagocytosis of FRD1 was significantly less than that of FRD1131 in both phagocytosis models (i.e., flow cytometry and gentamicin protection) in the presence of 0.5% serum, thus indicating that alginate plays a major role in the resistance of mucoid *Pseudomonas* to phagocytosis in general (**Figures 1E, F**).

### Flagellin expression contributes to phagocytosis of mucoid Pseudomonas aeruginosa

**Figure 2:**
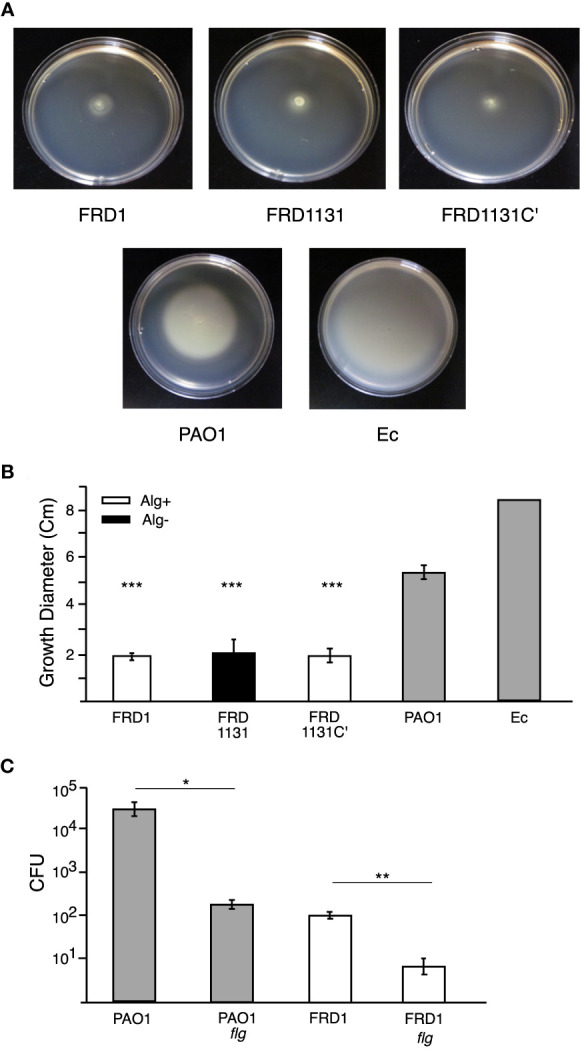
Motility of *P. aeruginosa* strains and effect of a flagella mutation. **(A)** Representative images of motility plates incubated 24 h after inoculation. **(B)** Soft (0.5%) agar plates were inoculated with FRD1, FRD1131, FRD1131C’, PAO1 or *E. coli* HfrH and incubated at 37°C for 24 h. The diameter of the resulting growth was measured. FRD1, FRD1131, and FRD1131C’ demonstrated a lack of motility relative to the motile PAO1 and HfrH. Data are shown as mean and standard deviation of 3 separate experiments. **(C)** Effect of flagellum mutation on phagocytosis. MH-S macrophages were infected with FRD1, PAO1 and their Flg- mutants for 1 h. CFU indicates the number of colony-forming units recovered from macrophages. Phagocytosis of Flg^-^ mutants was significantly decreased. Data are shown as mean and standard deviation of a representative triplicate experiment. Statistical significance was determined by ANOVA (* P < 0.05, ** P < 0.01, *** P < 0.001).

Both flagellum and motility are critical for promoting the phagocytosis of P. aeruginosa (Mahenthiralingam et al., 1994; Mahenthiralingam and Speert, 1995; Amiel et al., 2010; Lovewell et al., 2011). To ensure that the inhibitory effects of alginate production on phagocytosis were not due to just changes in motility, we compared the motility of the different strains of P. aeruginosa using a soft-agar plate method. Strain PAO1 (a well-studied wound isolate) was observed to be highly motile, as was E. coli HfrH, but the FRD strains (i.e., FRD1, FRD1131 and FRD1131C’) were essentially non-motile (**Figures 2A, B**). FRD1 and FRD1131 have a mucA mutation, which is known to activate expression of the gene for AmrZ, a repressor of motility (Tart et al., 2005; Tart et al., 2006). Thus, lack of motility in the FRD strains was to be expected. Our motility tests showed that the algD::Tn501 mutation in FRD1131 did not enhance its motility. To test the possibility that the expression of flagelum in FRD1 might still contribute to its phagocytosis, we constructed flgB::transposon insertion mutants to eliminate expression of flagellum. The flgB mutant of strain PAO1 was ~100-fold less susceptible to phagocytosis compared to the wild-type parent strain (**Figure 2C**), which was consistent with previous studies. Interestingly, the flgB mutant of FRD1 was ~10-fold less susceptible to phagocytosis than the parent strain, suggesting that this surface structure can still contribute to phagocytic uptake even in a mucoid mucA strain where flagella production is repressed.

### Bacterial expression of alginate is required for inhibition of phagocytosis

**Figure 3:**
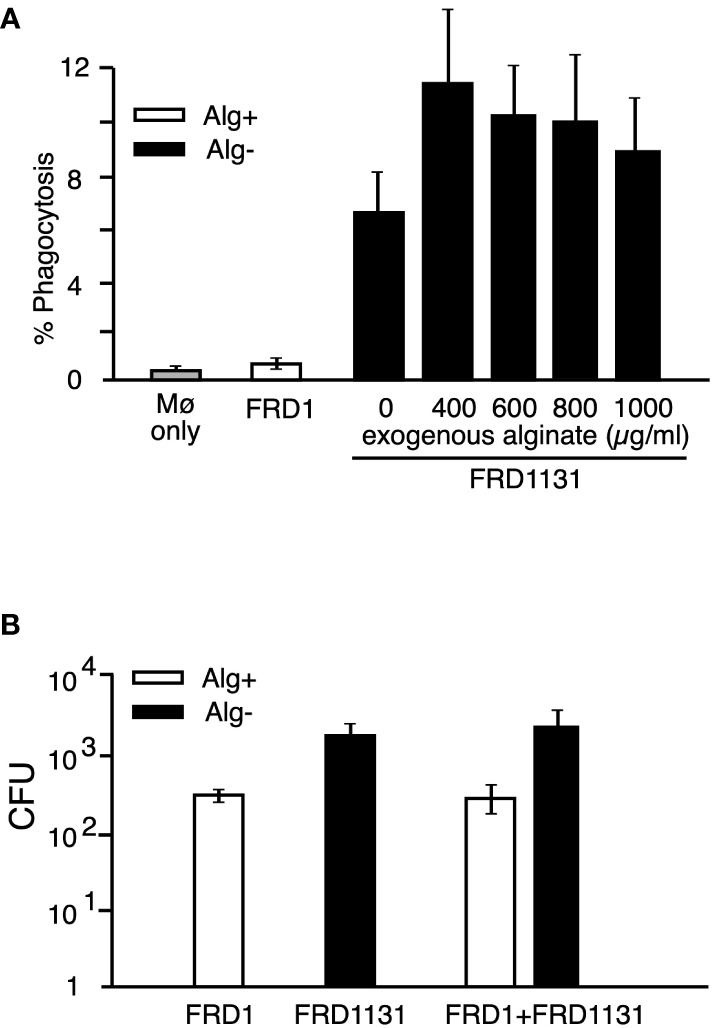
Effect of added alginate on the phagocytosis of nonmucoid *P. aeruginosa*. **(A)** MH-S macrophages were infected with GFP-expressing FRD1 or FRD1131 for 30 min in the presence of increasing concentrations of bacterial alginate. Percent phagocytosis indicates the percent of macrophages that are associated with bacteria as detected by flow cytometry. Phagocytosis of FRD1131 (Alg^-^) was not significantly inhibited by the addition of FRD1 alginate. Data are shown as mean and standard deviation of 3 separate experiments. **(B)** MH-S macrophages were infected with FRD1, FRD1131 or both strains combined for 1 h. CFU indicates the number of colony-forming units recovered from macrophages. Phagocytosis of FRD1131 was not significantly inhibited by the addition of FRD1. Data are shown as mean and standard deviation of three separate experiments.

To determine if alginate activates a signaling pathway that inhibits phagocytosis, we investigated the effect of alginate on the phagocytosis of a non-alginate expressing strain. MH-S macrophages were cultured with Alg^-^ FRD1131 in the presence of increasing concentrations of alginate. For these experiments the alginate was added with the FRD1131. However, the addition of alginate did not protect FRD1131 from phagocytosis when the alginate was purified from FRD1 (**Figure 3A**) or from brown seaweed (**Supplemental Figure S3**). This indicates that alginate does not activate an inhibitory pathway. To further address this issue, we determined whether the presence of Alg^+^ FRD1 protects Alg^-^ FRD1131 from phagocytosis. Since FRD1 and FRD1131 have visually distinct colony morphologies (i.e., mucoid vs. nonmucoid), it was straightforward to distinguish the strains in a mixed culture. When both strains were added to macrophages together, there was no significant difference between the phagocytosis of FRD1131 when mixed with FRD1 compared to FRD1131 alone (**Figure 3B**). Thus, alginate production by FRD1 was unable to inhibit the phagocytosis of FRD1131, suggesting that alginate only benefits the individual bacteria that produce it. Likewise, FRD1 was phagocytized equally regardless of the presence of FRD1131. Taken together these findings demonstrate that alginate does not deliver a negative signal, but rather blocks activation of signaling necessary for phagocytosis.

### Alginate production inhibits macrophage signaling cascades

**Figure 4:**
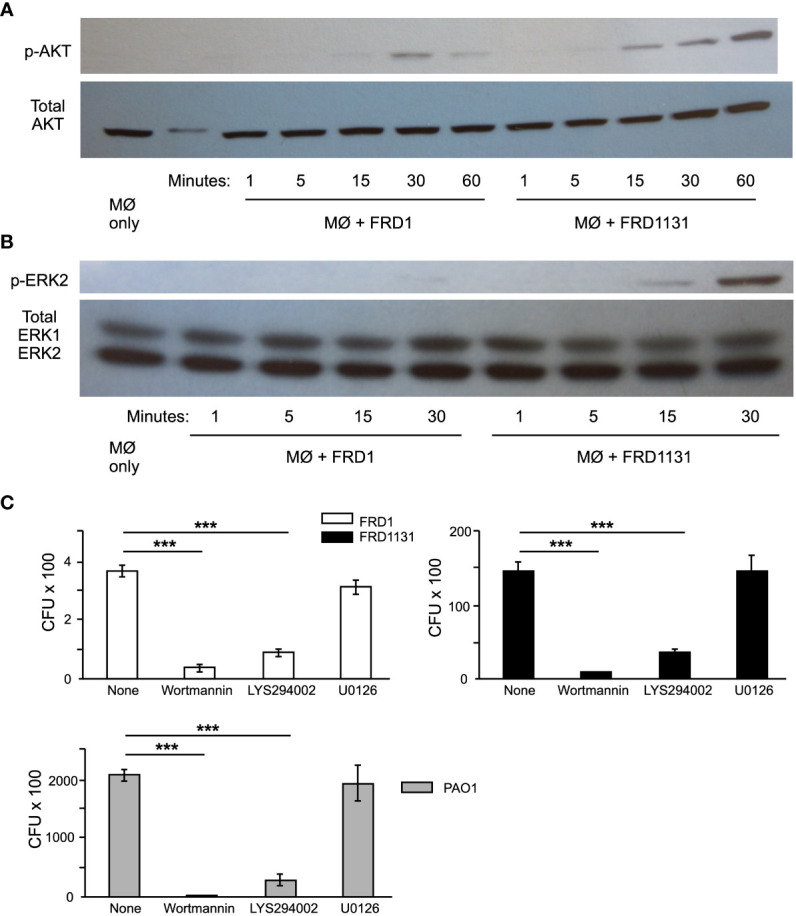
Effect of alginate production on intracellular signaling events. **(A)** Serum starved MH-S macrophages were infected with FRD1 or FRD1131 for 1 h. Cell lysates were collected and a Western blot was performed using anti-p-AKT and anti-pan-AKT antibodies. Infection with Alg^+^ FRD1 resulted in delayed AKT activation at 30 min compared to 15 min activation with FRD1131. Data shown is a representative triplicate experiment. **(B)** Serum starved MH-S macrophages were infected with FRD1 or FRD1131 for 30 min. Cell lysates were collected and a Western blot was performed using anti-p-ERK and anti-pan-ERK antibodies. Infection with Alg^+^ FRD1 resulted in decreased ERK activation at 30 min compared to Alg^-^ FRD1131. Data shown is a representative triplicate experiment. **(C)** To determine the effect AKT and ERK inhibitors on phagocytosis, MH-S macrophages were infected with FRD1 or FRD1131 for 1 h in the presence of wortmannin, LY294002, or U0126. CFU indicates the number of colony-forming units recovered from macrophages. Both AKT inhibitors, wortmannin and LY294002, significantly inhibited phagocytosis of FRD1, FRD1131 and PAO1. However, the ERK inhibitor U0126 was unable to inhibit phagocytosis of any strain tested. Data are shown as mean and standard deviation of a representative triplicate experiment. Statistical significance was determined by ANOVA (* P < 0.05, ** P < 0.01, *** P < 0.001).

The initial interaction between phagocytes and bacteria leading up to phagocytosis results in the activation of the phosphoinositide 3-kinase (PI3K) intracellular signaling pathway, which includes phosphorylation of AKT and ERK (Kannan et al., 2008). To explore the mechanism underlying alginate induced resistance to phagocytosis, the effect of FRD1 and FRD1131 infection on phosphorylation of AKT and ERK in MH-S macrophages were compared. Alg^-^ FRD1131 induced p-AKT in MH-S cells as early as 15 min post infection, whereas Alg^+^ FRD1-induced activation was both weak and delayed to 30 min (**Figure 4A**). The duration of AKT activation was also significantly lower with FRD1 compared to FRD1131 activated macrophages, which maintained activation at 60 min post infection (**Figure 4A**). Similar to AKT activation, infection with FRD1 resulted in delayed and decreased ERK2 activation compared to infection with FRD1131 (**Figure 4B**). While p-ERK2 was faintly detectable at 30 min in the FRD1 samples, FRD1131 induced ERK activation as early as 15 min post infection.

To confirm that AKT and ERK pathways are indeed important for the phagocytosis of *P. aeruginosa* by alveolar macrophages, the effect of specific pathway inhibitors on phagocytosis was evaluated. When MH-S macrophages were pretreated with wortmannin, LY294002, or U0126, inhibition of the activation of PI3 kinase or ERK1/2 was demonstrated (**Supplemental Figure S4**). Both AKT inhibitors, (wortmannin and LY294002) significantly inhibited phagocytosis of all *P. aeruginosa* strains tested (**Figure 4C**). However, the ERK inhibitor (U0126) did not inhibit phagocytosis of FRD1, FRD1131 or PAO1 (**Figure 4C**). Taken together these findings indicate that alginate production by *P. aeruginosa* inhibits interaction with macrophages, depressing PI3K signaling, which in turn leads to inhibition of phagocytosis.

### Alginate production inhibits the binding of P. aeruginosa to macrophage receptors

**Figure 5:**
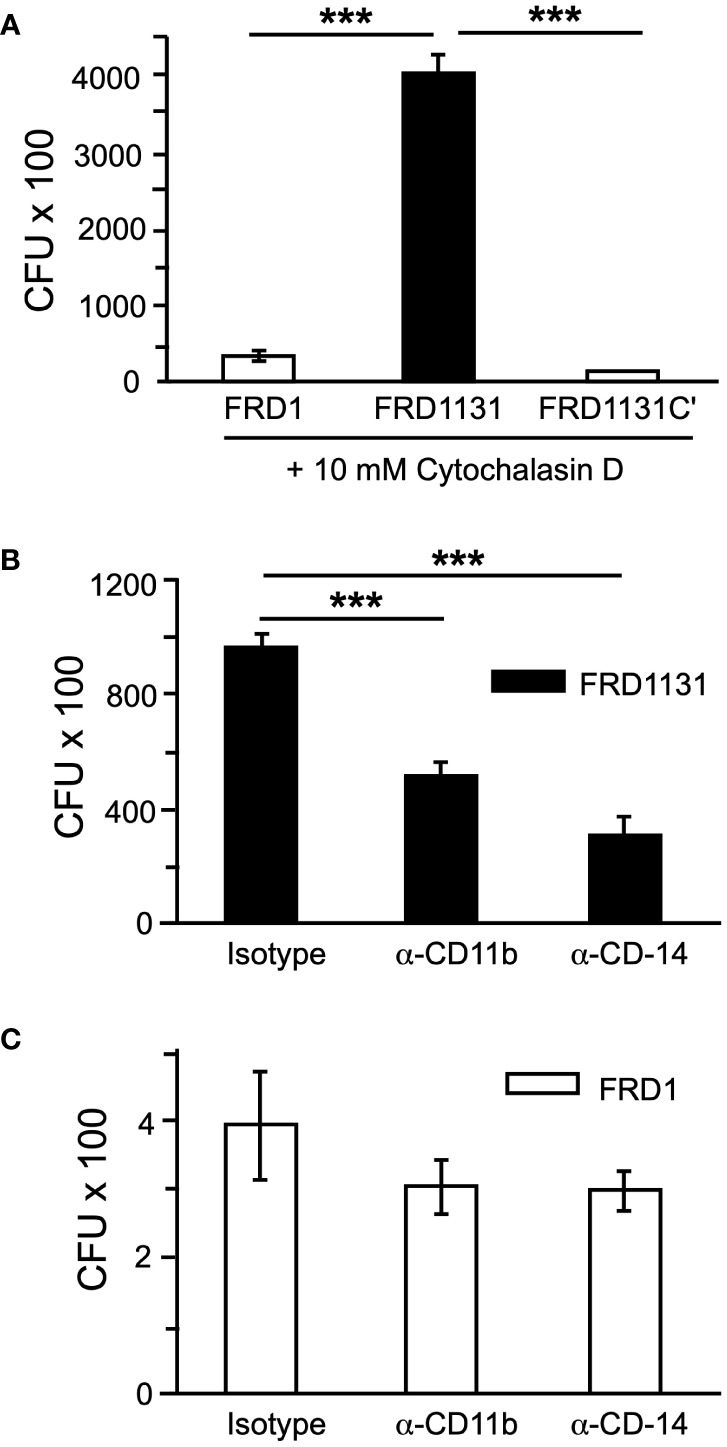
Alginate production blocked receptor-ligand binding. **(A)** To determine if alginate production affects tethering to macrophages, MH-S macrophages were treated with cytochalasin D to inhibit phagocytosis and infected with FRD1, FRD1131 or FRD1131C’ for 1 h. After incubation, macrophages were washed to remove unbound bacteria and lysed to free any bound bacteria. CFU indicates the number of colony-forming units recovered from macrophages. Binding of FRD1 and FRD1131C’ was significantly reduced compared to FRD1131. Data are shown as mean and standard deviation of a representative triplicate experiment. **(B, C)** To identify cell surface receptors that bind *P. aeruginosa* FRD, MH-S macrophages were treated with CD11b (Abcam, ab64347) or CD14 (Abcam, ab6083) blocking antibodies and infected with FRD1 **(B)** or FRD1131 **(C)** for 1 h. A rat IgG2b isotype control (BD Biosciences, 16061) was used which would presumably function similarly as a control for both the rat (CD11b) and murine (CD14) antibodies. CFU indicates the number of colony-forming units recovered from macrophages. Both antibodies significantly inhibited phagocytosis of FRD1131 compared to the isotype control. However, phagocytosis of FRD1 was not significantly inhibited by either antibody. Data are shown as mean and standard deviation of a representative triplicate experiment. Statistical significance was determined by ANOVA (* P < 0.05, ** P < 0.01, *** P < 0.001).

The first step in phagocytosis is the binding of bacterial ligands to receptors on the macrophage surface that tether the bacteria to the phagocyte. Since alginate, either exogenous or alginate produced in a mixed culture, was unable to inhibit phagocytosis, it was possible that alginate on the bacterial surface could interfere with binding to the macrophages and thus the activation of signaling pathways that are required for phagocytosis. To determine if alginate inhibits binding to macrophages, cytochalasin D was used to inhibit actin polymerization and prevent phagocytosis without affecting cell surface binding. Alg^+^ strains FRD1 and FRD1131C’ were shown to bind significantly less than Alg^-^ FRD1131 (**Figure 5A**), indicating that alginate production inhibits binding to macrophages.

Next, the effect of alginate expression on binding to specific cell surface receptors was explored. Both CD11b (known as CR3 in the mouse) and CD14 have been shown to be important for the phagocytosis of *P. aeruginosa* by macrophages and dendritic cells and to activate PI3 kinase signaling (Heale et al., 2001). Considering the above tethering results, the possibility was raised that alginate interfered with binding to either of these receptors. To address this, MH-S macrophages were pretreated with specific blocking antibodies or an isotype control before infection with FRD1 or FRD1131. When pretreated with α-CD11b(CR3) or α-CD14, phagocytosis of Alg^-^ FRD1131 was inhibited at least 2-fold (**Figure 5B**). In contrast, phagocytosis of Alg^+^ FRD1 was not significantly inhibited by the addition of receptor blocking antibodies (**Figure 5C**), suggesting that alginate interferes with binding to these receptors.

### Alginate expression does not affect chemokine production

**Figure 6:**
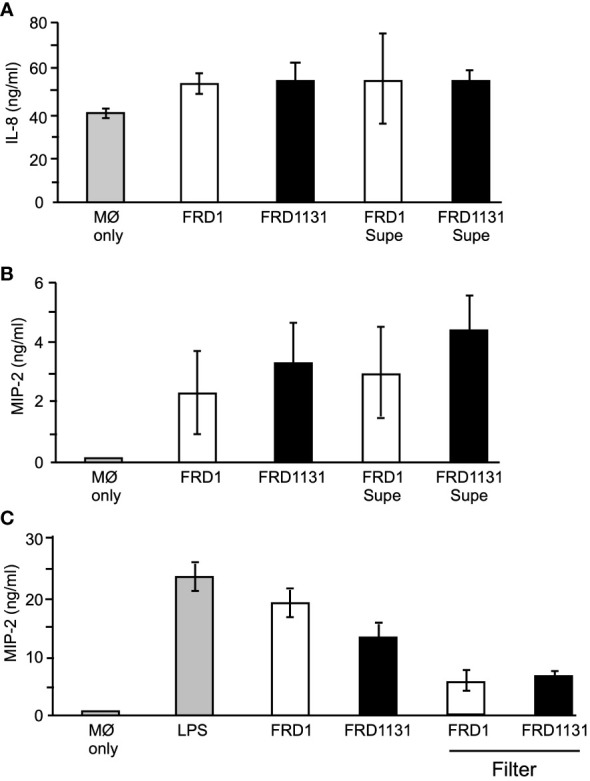
Effect of alginate production on IL-8 and MIP-2 production. **(A)** To determine the effect of alginate on IL-8 production, THP-1 macrophages were infected with FRD1 or FRD1131, or treated with FRD1 or FRD1131 supernatants (Supe) for 8 h. Supernatants were collected and analyzed by ELISA for IL-8 production. No significant difference was found between the amount of IL-8 induced by FRD1 compared to FRD1131, nor their supernatants. Data are shown as mean and standard deviation of 3 separate experiments. **(B)** To determine the effect of alginate on MIP-2 production, MH-S macrophages were infected with FRD1, FRD1131, or treated with FRD1 or FRD1131 supernatants for 8 h. Supernatants were collected and analyzed by ELISA for MIP-2 production. No significant difference was found in the amount of IL-8 induced by FRD1 compared to FRD1131, nor by their respective supernatants. Data are shown as mean and standard deviation of three separate experiments. **(C)** To examine contact dependence on MIP-2 production, MH-S macrophages were infected with FRD1 or FRD1131 either directly or above a filter for 8 h. Supernatants were collected and analyzed by ELISA for MIP-2 production. No significant difference was found between the amount of IL-8 induced by FRD1 compared to FRD1131, nor when separated by a filter (although the filters changed the volume which lowered the overall MIP-2 concentration). Data are shown as mean and ANOVA analysis of three separate experiments.

Cytokine production plays an important role in promoting lung damage in CF patients (Balough et al., 1995; Tabary et al., 1998; Brennan, 2008). Many neutrophils traffic to the lung, which leads to lung damage despite the inability to clear the infection. IL-8 is a chemokine produced by macrophage that functions as a major neutrophil chemoattractant. As shown above, mucoid *P. aeruginosa* inhibited AKT activation, which could affect downstream activation of NF-κB. Since NF-κB is responsible for IL-8 production, alginate expression could lead to reduced cytokine production in macrophages due to decreased receptor-ligand interactions (Yang and Jones, 2009). To determine if IL-8 production was affected by alginate production, the level of IL-8 in supernatants of THP-1 was measured 8 h post infection. Interestingly, there was no significant difference in the amount of IL-8 induced by Alg^+^ FRD1 compared to Alg^-^ FRD1131 (**Figure 6A**). This suggested that the majority of IL-8 produced by macrophages was contact independent. If this were the case, the inhibited binding due to alginate would not affect IL-8 production. To determine if contact with *P. aeruginosa* was necessary for IL-8 production, FRD1 and FRD1131 were cultured in RPMI for 8 h, supernatants were collected and then applied to THP-1 macrophages for an additional 8 h. As shown in **Figure 6A**, the *P. aeruginosa* supernatants of Alg^+^ and Alg^-^ strains were able to induce IL-8 in THP-1 macrophages. There was no significant difference in the amount of IL-8 induced by the supernatants compared to the amount induced by the *P. aeruginosa* strains themselves. Therefore, IL-8 production by THP-1 macrophages appeared to be contact independent and was unaffected by the production of alginate.

Murine macrophages do not produce IL-8 and instead produce a similar neutrophil chemoattractant called MIP-2. MH-S macrophages were cultured with FRD1 or FRD1131 for 8 h. As with IL-8 production, there was no significant difference in the amount of MIP-2 produced between FRD1 and FRD1131 (**Figure 6B**). Additional chemokines (e.g., monocyte CCL2) were not tested. Supernatants collected after eight hours of culture were tested to determine contact independence of MIP-2 production. There was no significant difference in MIP-2 production induced by the supernatants compared to the strains themselves (**Figure 6B**). A possible problem with the use of 8 h supernatants was that the soluble, stimulatory factors in the supernatants would be highly concentrated immediately, whereas the wells with the *P. aeruginosa* strains added directly would slowly build up to that high concentration over the 8 h incubation. As a more direct assessment of the contact dependence of MIP-2 production, 0.4 µm filter inserts were used to separate the added *P. aeruginosa* from the macrophages to allow soluble factors to diffuse throughout the media. As in the previous experiments, there was no significant difference in the amount of MIP-2 produced between Alg^+^ FRD1 and Alg^-^ FRD1131 with, or without, the filter (**Figure 6C**). However, the use of the filter reduced the amount of MIP-2 produced suggesting that MIP-2 production was due to soluble factors and somewhat independent of contact with the bacteria.

## Discussion

The goal of this research was to determine the mechanism by which alginate, an exopolysaccharide secreted by mucoid *P. aeruginosa*, inhibits their phagocytosis and destruction by macrophages as occurs in a biofilm. The mucoid phenotype is common to strains isolated from patients with CF and COPD that have chronic pulmonary disease with *P. aeruginosa*. The mucoid phenotype has been shown to enhance the survival of *P.* aeruginosa in the lung and significantly increase the morbidity and mortality of CF patients (Govan and Deretic, 1996; Sadikot et al., 2005). Mucoid *P. aeruginosa* is protected against non-opsonic phagocytosis in both the planktonic and biofilm modes of growth (Ruhen et al., 1980; Cabral et al., 1987; Krieg et al., 1988; Pier et al., 2001; Leid et al., 2005). Therefore, a better understanding of how alginate protects *P. aeruginosa* from phagocytosis could lead to the development of novel therapeutics for chronic pseudomonal infections. In this study we sought to quantitatively measure the effect of alginate production on the inhibition of non-opsonic phagocytosis of planktonic *P. aeruginosa*.

In initial experiments, the human monocytic cell line THP-1 was used as a model for phagocytosis. However, since alveolar macrophages are known to have characteristics that distinguish them from peripheral macrophages, we primarily used the murine alveolar macrophage cell line MH-S which is more similar to those present in the lung infection environment (Monick et al., 2004; Gwinn and Vallyathan, 2006). Nevertheless, comparable results were obtained with both macrophage cell lines. *P. aeruginosa* strain FRD1, a genetically manipulable mucoid CF clinical isolate (Ohman and Chakrabarty, 1981), was used to address the effect of alginate production (Alg^+^) on phagocytosis. FRD1131 is a non-mucoid isogenic *algD*::Tn*501-33* mutant (Chitnis and Ohman, 1993), and the site of the transposon was precisely mapped here. The Alg^+^ phenotype was readily restored to FRD1131 by genetic complementation. Since the only difference between these bacterial strains was the production of alginate, we could specifically examine the effect of alginate expression on phagocytosis. Both strains had the *mucA* mutation, which is typical in mucoid conversion (Martin et al., 1993), so both have the complex σ^22^ stress-response system activated (Wood et al., 2006; Wood and Ohman, 2009). Phagocytosis was assessed using two assays that measured different aspects of phagocytosis. The total number of bacteria that were phagocytized was determined using the gentamicin protection assay where macrophages were lysed and CFUs counted. The fraction of macrophages engaged in phagocytosis was determined by using GFP-expressing bacteria and flow cytometry. Initial experiments confirmed that Alg^+^ FRD1 was indeed significantly more resistant to opsonic and non-opsonic phagocytosis than its Alg^-^ mutant derivative, FRD1131.

Since flagella-mediated motility plays a major role in the effectiveness of phagocytic uptake of *P. aeruginosa* (Amiel et al., 2010; Lovewell et al., 2011), it was important that we also consider the role of the flagellum. Alginate production and flagellum expression are inversely expressed in *P. aeruginosa* when *mucA* mutation activates σ^22^ (Tart et al., 2005; Tart et al., 2006). Consistent with this, both FRD1 and FRD1131, which have a *mucA* mutation, appeared to be non-motile under laboratory conditions. Interestingly, flagellar motility and not just the flagellum itself appears to be responsible for the higher rate of phagocytosis of *P. aeruginosa* (Amiel et al., 2010; Lovewell et al., 2011). Flagellar motility is also critical for internalization, but not binding to phagocytic cells (Mahenthiralingam et al., 1994; Mahenthiralingam and Speert, 1995). Interestingly, we observed a 10-fold reduction in phagocytosis when flagellum expression in FRD1 was removed by mutation. However, the same flagella mutation in *mucA*+ PAO1 resulted in a 100-fold reduction in the rate of phagocytosis. These results suggests that flagellar motility was at least transiently expressed by FRD strains under the conditions examined here despite the down regulation of flagellum expression that occurs when σ^22^ is activated due to *mucA* mutation and the apparent non-motility observed. In that both Alg^+^ FRD1 and Alg^-^ FRD1131 appear to be equivalent with respect to flagellar motility, then it was reasonable to assume their differences in phagocytic susceptibility is due to alginate and not motility. It is also worth noting that differences in the macrophages and *P. aeruginosa* strains utilized could affect the analysis of motility and phagocytosis. In this study we used human and murine macrophage cell lines, whereas previous studies used human peripheral blood monocyte-derived macrophages that may express different amounts of TLR5, which is a receptor for flagellum.

Previous studies suggest that exogenous alginate may protect planktonic and biofilm *P. aeruginosa* from phagocytic killing (Ruhen et al., 1980; Leid et al., 2005), raising the possibility that alginate activates a signaling pathway that inhibits phagocytosis. However, when cultured together, alginate produced by FRD1 was unable to inhibit the phagocytosis of Alg^-^ FRD1131. This implied that alginate did not induce a negative signal within the macrophages because the presence of alginate in the media would have inhibited phagocytosis if that were the case. Also, alginate added exogenously even at high levels (1 mg ml^-1^) was unable to protect nonmucoid *P. aeruginosa* from phagocytosis. Again, this indicated that alginate was not negatively regulating macrophages or else this would have inhibited the phagocytosis of Alg^-^ FRD1131. This suggested that expression of alginate on the bacterial surface interfered with a receptor-ligand interaction that is necessary for promoting phagocytosis.

The initial interaction between phagocytes and bacteria involves multiple receptors and the activation of signaling cascades. Previous studies have implicated both the PI3K and MAPK pathways in phagocytosis (Kannan et al., 2008). Furthermore, CD11b (i.e., CR3 in mice) and CD14, which are implicated in uptake of *P. aeruginosa*, can contribute to PI3K signaling (Thieblemont et al., 1995; Medvedev et al., 1998; Heale et al., 2001; Sun et al., 2010), which is one of the multiple signaling pathways implicated in phagocytosis (Flannagan et al., 2012). Thus, to explore the mechanism underlying alginate induced resistance to phagocytosis, the effect of FRD1 and FRD1131 infection on the down-stream phosphorylation of AKT and ERK in macrophages was compared. The results showed that soon after infection, Alg^-^ FRD1131 induced p-AKT and p-ERK. However, by comparison, Alg^+^ FRD1 showed reduced activation and it was delayed. The addition of AKT inhibitors significantly inhibited the phagocytosis of *P. aeruginosa*, confirming that this pathway is indeed important for the phagocytosis of *P. aeruginosa* by alveolar macrophages. Overall, these findings suggest that alginate production by *P. aeruginosa* blocks phagocyte-bacterial interaction, which depresses PI3K signaling and leads to inhibition of phagocytosis.

The first step in phagocytosis is the binding of bacterial ligands to receptors on the macrophage surface, which tethers the bacteria to the phagocyte. Since exogenous alginate or alginate produced in a mixed culture were unable to inhibit phagocytosis, it was possible that alginate on the bacterial surface could interfere with binding to the macrophages and thus the activation of signaling pathways that are required for phagocytosis. To determine if alginate inhibits binding to macrophages, cytochalasin D was used to inhibit actin polymerization and therefore prevent phagocytosis, but the drug would not affect cell surface binding. Alg^+^ strains FRD1 and FRD1131C’ were shown to bind significantly less than the Alg^-^ FRD1131, suggesting that alginate production inhibits binding to macrophages. To identify receptors that may be blocked by alginate, we investigated the contribution of CD11b and CD14, which are cell surface receptors that have been reported to facilitate the phagocytosis of *P. aeruginosa* (Heale et al., 2001). CR3(CD11b) has been shown to bind to a number of ligands associated with *P. aeruginosa* including LPS, APG, and β-glucan (Brown, 2006; Zhou et al., 2013). Blocking either CD11b or CD14 with specific antibodies led to a significant decrease in phagocytosis of non-mucoid *P. aeruginosa* by murine macrophages, indicating that these receptors play a role in the uptake of strain FRD. However, this inhibitory effect was not significant with mucoid *P. aeruginosa*, suggesting that both of these receptors were being blocked by alginate and that further blockade with the antibodies was ineffective. CD11b and CD14 can activate PI3K and MEK1/2. CD14/TLR4 signaling through MyD88, IRAK1/4, and TRAF6 activate PI3K and MEK1/2 (Schepetkin and Quinn, 2006). CR3 can also lead to PI3K and MEK activation through PLC and protein kinase C (PKC) (Schepetkin and Quinn, 2006; Hajishengallis, 2010; Underhill and Goodridge, 2012). AKT and ERK phosphorylation were used to measure PI3K and MEK1/2 activation. Both ERK and AKT activation were decreased by alginate production. While PI3K activation was found to be critical for phagocytosis of *P. aeruginosa*, loss of MEK activation had no significant effect. However, reduced ERK activation by alginate may lead to altered inflammatory signaling despite not being necessary for phagocytosis.

Interestingly, we found no significant difference in the amount of IL-8 or MIP-2 induced by Alg^+^ and Alg^-^ *P. aeruginosa*. This could be because MIP-2 induction was found to be at least partially contact-independent, which is often the case in cytokine induction (Underhill and Goodridge, 2012). It is possible that while IL-8 and MIP-2 production are NF-κB dependent, NF-κB may be sufficiently activated despite reduced AKT signaling. It is also possible that NF-κB activity is stimulated through a PI3K-independent pathway, which could be accomplished through other scavenger and pattern recognition receptor signaling.

In summary, this study demonstrated for the first time that alginate on the bacterial surface blocks binding of the bacteria to macrophages *via* CD14 and CR3 causing decreased intracellular signaling and reduced phagocytosis. Interestingly, the decreased binding did not appear to inhibit chemokine production. Inhibition of phagocytosis while maintaining cytokine production may exacerbate lung inflammation and subsequent tissue destruction by recruiting more neutrophils to the lung environment without clearing the infection. These findings argue that therapies specifically designed to either interfere with alginate production or enhance phagocytosis could prove useful in the treatment of chronic lung infections by mucoid *P. aeruginosa*.

### Experimental procedures

#### Bacterial culture media and growth conditions

*P. aeruginosa* and *Escherichia coli* strains were routinely grown in L broth (10 g tryptone, 5 g yeast extract, 5 g NaCl per liter), which was also solidified with 1.5% agar for L agar plates. Strains were grown routinely in L broth at 37°C with shaking. Antibiotics were included when necessary for plasmid maintenance at the following concentrations for *P. aeruginosa*: ampicillin, 100 µg ml^-1^; carbenicillin, 150 µg ml^-1^; kanamycin, 30 µg ml^-1^; and tetracycline, 100 µg ml^-1^.

#### Bacterial strains and plasmids

The bacterial strains and plasmids used in this study are shown in **Table 1**. P*. aeruginosa* FRD1 is a CF clinical isolate in which alginate production is constitutively active due to a *mucA22* mutation. FRD1131 is an isogenic mutant of FRD1 that contains a Tn*501-33* insertion mapped to the *algD* promoter; *algD* is the first gene in the alginate operon and the transposon is polar on all downstream genes, so FRD1131 is unable to produce alginate. pALG2 contains the entire *algD* operon and complemented FRD1131 back to the mucoid phenotype by integrating the plasmid (of narrow host range) into the chromosome by homologous recombination. PAO1 is a clinical wound isolate that does not produce alginate. GFP-expressing strains were constructed by transferring pMF230 into the recipient strain by conjugation using the conjugative helper plasmid pRK2013.

**Table 1:** Bacterial strains and plasmids used in this study.

Name	Genotype/Phenotype	Source
*P. aeruginosa* strains		
FRD1	*mucA22* Alg^+^	(Ohman et al., 1981)
FRD1131	*mucA22 algD*::Tn*501* Alg^-^	(Chitnis et al., 1993)
FRD1131C’	FRD1131(pALG2) Cb^R^ Alg^+^	This study
FRD1 Flg^-^	FRD1 *flgB*::ISphoA/hah Tc^R^ Flg^-^	This study
PAO1	Wild-type wound isolate Alg^-^	(Holloway et al., 1979)
PAO1 Flg^-^	PAO1 *flgB* (PA1077)::ISphoA/hah Tc^R^ Flg^-^	(Held et al., 2012)
Plasmids		
pMF230	Broad-host-range GFP^+^ Ap/Cb^R^	(Nivens et al., 2001)
pRK2013	*oriV*(ColE1) Tra^+^ Km^R^	This laboratory
pALG2	*oriV*(ColE1) *oriT algD* operon Ap/Cb^R^	(Chitnis et al., 1993)
pDONR221	Gateway cloning vector Km^R^	Invitrogen

Phenotypic abbreviations: Alg+, alginate over-production; Flg-, loss of flagella; GFP, expression of green fluorescent protein, Tra+, transfer of plasmids via a conjugative apparatus. Antibiotic resistances: Tc, tetracycline; Cb, carbenicillin; Ap, ampicillin; Km, kanamycin.

### Determination of the site of Tn*501-33* in FRD1131

The site of the Tn*501-33* insertion in FRD1131 was previously determined to be at *algD* by restriction mapping analysis (Chitnis and Ohman, 1993). To determine the precise location of the transposon, inverse PCR was used. FRD1131 chromosomal DNA was digested with SalI, which cuts 1886 bp from one end of Tn*501*. The DNA fragments of the SalI digest were purified (QIAquick PCR purification kit) and then circularized with T4 DNA ligase. Primers specific for the ends of the Tn*501* sequences (bold) with Gateway cloning sequences (GGGGACAAGTTTGTACAAAAAAGCAGGCT-ctacgattgaccgcagtaag, GGGGACCACTTTGTACAAGAAAGCTGGGT-caaagagctgtcacgagaac) were used to amplify the DNA containing the junction between Tn*501* and chromosomal DNA, which was cloned into pDONR221 with BP Clonase II (Invitrogen) and transformed into *E. coli* DH5α. The sequence analysis of this plasmid showed that Tn*501-33* was located 16 bp downstream of the *algD* start of transcription and 351 bp upstream of the *algD* start codon.

#### Strain construction

A PAO1 library of sequence-defined (tetracycline-resistant) transposon Tn5-related insertions (Held et al., 2012) was purchased from The University of Washington. A specific transposon was moved into the FRD1 chromosome by phage transduction using phage F116L. Briefly, plate lysates were made on the PAO1 library mutant, and the filter-sterilized lysate was incubated for 4 h with the recipient strain and then plated onto L-agar with tetracycline (100 µg ml^-1^) to select for inheritance of the transposon by homologous recombination.

#### Macrophage culture

Human THP-1 and murine MH-S macrophages were purchased from the American Type Culture Collection (TIB-202 and CRL-2019, respectively) and grown in complete RPMI containing 10% fetal bovine serum (Gibco), 100 U/ml penicillin and streptomycin, and 2 mM L-glutamine. Cells were counted by hemocytometer prior to plating for each experiment. THP-1 cells were differentiated into phagocytes with 12-O-tetradecanoylphorbol-13-acetate (TPA) (Sigma, final concentration 3.2 x 10^-7^ M) for 2 days prior to infection experiments (Tsuchiya et al., 1982).

#### Flow cytometry-based phagocytosis assay

Sterile 12-well plates were seeded with 2 x 10^5^ cells of THP-1 or MH-S macrophages in RPMI with serum and antibiotics and incubated at 37°C in 5% CO_2_ for 30 min to become adherent to plates. Overnight cultures of GFP-expressing bacterial strains were diluted 1:10 in L broth and grown for ~2 h to mid-log phase growth at 5 x 10^8^ CFU ml^-1^ (OD_600_ 0.8). Bacterial cultures were added (10 µl) to the macrophages at an MOI of 50:1 and incubated at 37°C in 5% CO_2_ for 30 min. Media was removed from the adherent macrophage cultures, which were then gently washed with phosphate buffered saline (PBS) and harvested with phosphate buffered saline (PBS) + 0.02% EDTA. The cells were analyzed by flow cytometry (VCU Flow Cytometry Core Facility) to determine the percentage of cells with internalized GFP-expressing bacteria. Macrophage gating was determined first by forward scatter versus side scatter to eliminate dead cells and debris. Secondary gating was determined by GFP expression versus red autofluorescence to count the number of macrophages containing GFP-expressing bacteria (Pils et al., 2006).

#### Gentamicin protection assay

Phagocytosis of bacteria was evaluated by their protection from gentamicin as previously described (Fleiszig et al., 1997). Briefly, 2 x 10^5^ THP-1 or MH-S macrophages were grown in 12-well plates prior to infection. Macrophages were infected with different bacterial strains at an MOI of 50:1 as described above, and incubated at 37°C in 5% CO_2_ for 1 h. MH-S cells were washed with cold PBS, treated with gentamicin (2 mg ml^-1^) for 30 min to kill extracellular bacteria, washed twice with cold PBS, and subsequently lysed with 0.25% SDS to release intracellular bacteria. Controls included wells containing only bacteria treated with gentamicin (2 mg ml^-1^) for 30 min or 0.25% SDS, which resulted in 0% or 100% CFU, respectively, compared to the mixed wells. Cell lysates were serially diluted 1:10 and 100 µl of the dilutions were spread on L agar plates. Plates were incubated at 37°C overnight and colonies were counted to determine CFU. In some experiments, blocking antibodies were added 15 min prior to infection and signaling inhibitors were added 30 min prior to infection.

#### Motility assay

Flagella mediated motility was determined as previously described (Luzar et al., 1985). Briefly, overnight cultures of bacterial strains were diluted 1:10 and grown for ~2 h until mid-log phase growth (OD_600_ 0.8). Soft L agar plates (0.5% agar) were inoculated at one central point with 1 µl of the mid-log phase bacterial culture. After incubating at 37°C for 24 h, the diameters of the resulting growth were measured.

#### Alginate isolation and purification

Alginate secreted by *P. aeruginosa* FRD1 was partially purified as previously described (Franklin et al., 1994). Briefly, 1 ml of an overnight culture of alginate producing strain FRD1 was inoculated into 100 ml of L broth incubated overnight at 37°C with aeration. The culture was then centrifuged at 16,264 x *g* at 5°C for 60 min, and the supernatant was mixed with 2 vol of cold ethanol. The alginate precipitate was removed with a glass rod, placed in a petri dish, dried at 37°C and dissolved in 100 ml of 1 M NaCl at 5°C with agitation for 16 h. 100 ml of cold isopropanol was added to precipitate the alginate, and the mixture was centrifuged at 10,000 RPM at 5°C for 60 min. The pellet was dissolved in 20 ml saline by shaking overnight at 37°C. Once dissolved, contaminating proteins were removed by incubation with trypsin (0.5 mg ml^-1^) for 2 h at 37°C with shaking. 1 g of NaCl was dissolved in the mixture followed by the addition of 1 vol of cold isopropanol. The solution was centrifuged at 16,264 x *g* at 5°C for 60 min. The resulting precipitate was dissolved in 20 ml saline and dialyzed against distilled water. Alginate concentration was determined spectrophotometrically as previously described (Knutson and Jeanes, 1968) using seaweed alginate (Sigma) as a standard.

#### Western blot analysis

2 x 10^6^ MH-S macrophages per well were cultured in 6-well plates, serum starved for 6 h and infected at an MOI of 50:1 as described above. After infection, macrophages were washed twice with cold PBS and lysed with 70 µl RIPA (Cell Signal) containing 1 mM PMSF and supernatants were frozen at -80°C prior to analysis. Proteins (20 µg) were separated by SDS-PAGE and transferred to PVDF membranes using a semi-dry transfer cell (Bio-Rad) as described (Miller et al., 2008). The membrane was blocked with 2% BSA in TBS/Tween (0.05%, Amresco K873) for 1 h. Primary antibodies (anti-Phospho-Akt, anti-Akt, anti-Phospho-p44/42 MAPK, anti-p44/42 MAPK) (Cell Signal, 4060, 4691, 4370, 4695) were added at 1:1000 in TBS/Tween with 2% BSA at 4°C with shaking overnight. The membrane was washed 3 times, for 10 min each, with TBS/Tween and incubated with secondary antibody (α-Rabbit IgG-peroxidase, Sigma A9169) at 1:5000 in TBS/Tween with 2% BSA for 45 minutes at room temperature with shaking. The blot was washed 4 times, for 10 min each, with TBS/Tween. West Pico chemiluminescence substrate (Thermo Sci 34080) was added for 5 min prior to film exposure. To strip the blot to allow for additional probes, stripping buffer (Thermo Sci 21059) was added for 15 min at room temperature with shaking. The blot was washed with TBS/Tween and blocked with 2% BSA for 1 h.

#### IL-8 and MIP-2 ELISA

2 x 10^5^ THP-1 or MH-S macrophages were grown in 12-well plates for 16 h, infected with different bacterial strains at an MOI of 50:1 and incubated at 37°C in 5% CO_2_ for 8 h. Supernatants were collected and centrifuged to remove any remaining bacteria. IL-8 or MIP-2 levels were determined by ELISA using Quantikine kits (R&D Systems d8000c, mm200) as described by the manufacturer.

## Data availability statement

The original contributions presented in the study are included in the article/**Supplementary Material**. Further inquiries can be directed to the corresponding author.

## Author contributions

WR conducted all the experiments described as part of his PhD dissertation. DL and DO were co-advisors in the project. All authors contributed to the article and approved the submitted version.

## Supplementary material

Supplementary Figure 1 Time course of phagocytosis of FRD1 and FRD1131 by MH-S macrophages. **(A)** MH-S macrophages were infected with GFP-expressing Alg^+^ FRD1 or Alg^-^ FRD1131 for various time points up to 1 h. Percent phagocytosis indicates the percent of macrophages that are associated with GFP-expressing *P. aeruginosa* as detected by flow cytometry. Reduced phagocytosis of FRD1 compared to FRD1131 was observed as early as 30 min. Data represent one typical experiment of several performed. **(B)** Visualization of THP-1 macrophages following phagocytosis of GFP-expressing FRD1 and FRD1131. Images show GFP-expressing *P. aeruginosa* overlaid on a visible light image of THP-1 macrophages. THP-1 macrophages were infected with GFP-expressing FRD1 or FRD1131 for 30 min and visualized by fluorescent microscopy. Inhibited phagocytosis, and association, was observed with FRD1 compared to FRD1131.

Supplementary Figure 2 Percentage of MH-S macrophages that have phagocytized FRD1 or FRD1131 with increasing concentrations of human serum. MH-S macrophages were infected with GFP-expressing FRD1 or FRD1131 for 30 min in the presence of increasing concentrations of human serum. % Phagocytosis indicates the percent of macrophages that are associated with GFP-expressing *P. aeruginosa* as detected by flow cytometry. A substantial difference in the phagocytosis of FRD1 compared to FRD1131 was observed beginning at 0.5% human serum. Data represent the results of 1 of 3 typical experiments.

Supplementary Figure 3 Percentage of MH-S macrophages that have phagocytized FRD1131 after 30 min in the presence of seaweed alginate. MH-S macrophages were infected with GFP-expressing FRD1131 for 30 min with increasing concentrations of alginate from brown seaweed. % Phagocytosis indicates the percent of macrophages that are associated with GFP-expressing bacteria as detected by flow cytometry. Phagocytosis of Alg^-^ FRD1131 was not significantly inhibited by the addition of seaweed alginate. Data are shown as mean and standard deviation of three samples.

Supplementary Figure 4 Western blots showing p-ERK2, total ERK1/ERK2, p-AKT, and total AKT from MH-S macrophages after phagocytosis of FRD1 or FRD1131 in the presence of inhibitors. **(A)** To demonstrate AKT inhibition, serum starved MH-S macrophages treated with wortmannin or LY294002 and infected with Alg^-^ FRD1131 for 30 min. Cell lysates were collected and a Western blot was performed using anti-p-AKT or anti-pan-AKT antibodies. Both wortmannin and LY294002 successfully inhibited AKT activation. **(B)** To demonstrate ERK inhibition, serum starved MH-S macrophages treated with U0126 and infected with Alg^-^ FRD1131 for 30 min. Cell lysates were collected and a Western blot was performed using anti-p-ERK, and anti-pan-ERK antibodies. Despite low baseline phosphorylation, U0126 was able to inhibit ERK activation.

## References

[B1] Amiel E. Lovewell R. R. O'Toole G. A. Hogan D. A. Berwin B. (2010). Pseudomonas aeruginosa evasion of phagocytosis is mediated by loss of swimming motility and is independent of flagellum expression. Infect. Immun. 78, 2937–2945. doi: 10.1128/IAI.00144-10 20457788 PMC2897393

[B2] Balough K. McCubbin M. Weinberger M. Smits W. Ahrens R. Fick R. (1995). The relationship between infection and inflammation in the early stages of lung disease from cystic fibrosis. Pediatr. Pulmonol 20, 63–70. doi: 10.1002/ppul.1950200203 8570304

[B3] Brennan S. (2008). Innate immune activation and cystic fibrosis. Paediatric Respir. Rev. 9, 271–279, quiz 279–280. doi: 10.1016/j.prrv.2008.05.008 19026368

[B4] Brown G. D. (2006). Dectin-1: a signalling non-TLR pattern-recognition receptor. Nat. Rev. Immunol. 6, 33–43. doi: 10.1038/nri1745 16341139

[B5] Cabral D. A. Loh B. A. Speert D. P. (1987). Mucoid pseudomonas aeruginosa resists nonopsonic phagocytosis by human neutrophils and macrophages. Pediatr. Res. 22, 429–431. doi: 10.1203/00006450-198710000-00013 2891100

[B6] Chitnis C. E. Ohman D. E. (1993). Genetic analysis of the alginate biosynthetic gene cluster of *Pseudomonas aeruginosa* shows evidence of an operonic structure. Mol. Microbiol. 8, 583–590. doi: 10.1111/j.1365-2958.1993.tb01602.x 7686997

[B7] Flannagan R. S. Jaumouille V. Grinstein S. (2012). The cell biology of phagocytosis. Annu. Rev. Pathol. 7, 61–98. doi: 10.1146/annurev-pathol-011811-132445 21910624

[B8] Fleiszig S. M. Wiener-Kronish J. P. Miyazaki H. Vallas V. Mostov K. E. Kanada D.. (1997). Pseudomonas aeruginosa-mediated cytotoxicity and invasion correlate with distinct genotypes at the loci encoding exoenzyme s. Infect. Immun. 65, 579–586. doi: 10.1128/iai.65.2.579-586.1997 9009316 PMC176099

[B9] Folkesson A. Jelsbak L. Yang L. Johansen H. K. Ciofu O. Hoiby N.. (2012). Adaptation of pseudomonas aeruginosa to the cystic fibrosis airway: an evolutionary perspective. Nat. Rev. Microbiol. 10, 841–851. doi: 10.1038/nrmicro2907 23147702

[B10] Franklin M. J. Chitnis C. E. Gacesa P. Sonesson A. White D. C. Ohman D. E. (1994). *Pseudomonas aeruginosa* AlgG is a polymer level alginate C5-mannuronan epimerase. J. Bacteriol. 176, 1821–1830. doi: 10.1128/jb.176.7.1821-1830.1994 8144447 PMC205283

[B11] Govan J. R. W. Deretic V. (1996). Microbial pathogenesis in cystic fibrosis: mucoid *Pseudomonas aeruginosa* and. Burkholderia cepacia. Microbiol. Rev. 60, 539–574. doi: 10.1128/mr.60.3.539-574.1996 8840786 PMC239456

[B12] Gwinn M. R. Vallyathan V. (2006). Respiratory burst: role in signal transduction in alveolar macrophages. J. Toxicol. Environ. Health Part B Crit. Rev. 9, 27–39. doi: 10.1080/15287390500196081 16393868

[B13] Hajishengallis G. (2010). Complement and periodontitis. Biochem. Pharmacol. 80, 1992–2001. doi: 10.1016/j.bcp.2010.06.017 20599785 PMC2955993

[B14] Heale J. P. Pollard A. J. Stokes R. W. Simpson D. Tsang A. Massing B.. (2001). Two distinct receptors mediate nonopsonic phagocytosis of different strains of pseudomonas aeruginosa. J. Infect. Dis. 183, 1214–1220. doi: 10.1086/319685 11262203

[B15] Held K. Ramage E. Jacobs M. Gallagher L. Manoil C. (2012). Sequence-verified two-allele transposon mutant library for pseudomonas aeruginosa PAO1. J. Bacteriol 194, 6387–6389. doi: 10.1128/JB.01479-12 22984262 PMC3497512

[B16] Holloway B. W. Krishnapillai V. Morgan A. F. (1979). Chromosomal genetics of *Pseudomonas* . Microbiol. Rev. 43, 73–102. doi: 10.1128/mr.43.1.73-102.1979 111024 PMC281463

[B17] Kannan S. Audet A. Huang H. Chen L. J. Wu M. (2008). Cholesterol-rich membrane rafts and Lyn are involved in phagocytosis during pseudomonas aeruginosa infection. J. Immunol. 180, 2396–2408. doi: 10.4049/jimmunol.180.4.2396 18250449

[B18] Knutson C. A. Jeanes A. (1968). A new modification of the carbazole analysis: application to heteropolysaccharides. Anal. Biochem. 24, 470–481. doi: 10.1016/0003-2697(68)90154-1 5723302

[B19] Krieg D. P. Helmke R. J. German V. F. Mangos J. A. (1988). Resistance of mucoid pseudomonas aeruginosa to nonopsonic phagocytosis by alveolar macrophages *in vitro* . Infect. Immun. 56, 3173–3179. doi: 10.1128/iai.56.12.3173-3179.1988 3141284 PMC259720

[B20] Leid J. G. Willson C. J. Shirtliff M. E. Hassett D. J. Parsek M. R. Jeffers A. K. (2005). The exopolysaccharide alginate protects pseudomonas aeruginosa biofilm bacteria from IFN-gamma-mediated macrophage killing. J. Immunol. 175, 7512–7518. doi: 10.4049/jimmunol.175.11.7512 16301659

[B21] Lovewell R. R. Collins R. M. Acker J. L. O'Toole G. A. Wargo M. J. Berwin B. (2011). Step-wise loss of bacterial flagellar torsion confers progressive phagocytic evasion. PloS Pathog. 7, e1002253. doi: 10.1371/journal.ppat.1002253 21949654 PMC3174259

[B22] Luzar M. A. Thomassen M. J. Montie T. C. (1985). Flagella and motility alterations in pseudomonas aeruginosa strains from patients with cystic fibrosis: relationship to patient clinical condition. Infect. Immun. 50, 577–582. doi: 10.1128/iai.50.2.577-582.1985 3932214 PMC261995

[B23] Mahenthiralingam E. Campbell M. E. Speert D. P. (1994). Nonmotility and phagocytic resistance of pseudomonas aeruginosa isolates from chronically colonized patients with cystic fibrosis. Infect. Immun. 62, 596–605. doi: 10.1128/iai.62.2.596-605.1994 8300217 PMC186146

[B24] Mahenthiralingam E. Speert D. P. (1995). Nonopsonic phagocytosis of pseudomonas aeruginosa by macrophages and polymorphonuclear leukocytes requires the presence of the bacterial flagellum. Infect. Immun. 63, 4519–4523. doi: 10.1128/iai.63.11.4519-4523.1995 7591095 PMC173644

[B25] Martin D. W. Schurr M. J. Mudd M. H. Govan J. R. W. Holloway B. W. Deretic V. (1993). Mechanism of conversion to mucoidy in *Pseudomonas aeruginosa* infecting cystic fibrosis patients. Proc. Natl. Acad. Sci. U.S.A. 90, 8377–8381. doi: 10.1073/pnas.90.18.8377 8378309 PMC47359

[B26] Medvedev A. E. Flo T. Ingalls R. R. Golenbock D. T. Teti G. Vogel S. N.. (1998). Involvement of CD14 and complement receptors CR3 and CR4 in nuclear factor-kappaB activation and TNF production induced by lipopolysaccharide and group b streptococcal cell walls. J. Immunol. 160, 4535–4542. doi: 10.4049/jimmunol.160.9.4535 9574560

[B27] Miller A. V. Alvarez S. E. Spiegel S. Lebman D. A. (2008). Sphingosine kinases and sphingosine-1-phosphate are critical for transforming growth factor beta-induced extracellular signal-regulated kinase 1 and 2 activation and promotion of migration and invasion of esophageal cancer cells. Mol. Cell. Biol. 28, 4142–4151. doi: 10.1128/MCB.01465-07 18426913 PMC2423114

[B28] Monick M. M. Mallampalli R. K. Bradford M. McCoy D. Gross T. J. Flaherty D. M.. (2004). Cooperative prosurvival activity by ERK and akt in human alveolar macrophages is dependent on high levels of acid ceramidase activity. J. Immunol. 173, 123–135. doi: 10.4049/jimmunol.173.1.123 15210766

[B29] Murphy T. F. (2009). Pseudomonas aeruginosa in adults with chronic obstructive pulmonary disease. Curr. Opin. pulmonary Med. 15, 138–142. doi: 10.1097/MCP.0b013e328321861a 19532029

[B30] Nivens D. E. Ohman D. E. Williams J. Franklin M. J. (2001). Role of alginate and its O acetylation in formation of *Pseudomonas aeruginosa* microcolonies and biofilms. J. Bacteriol. 183, 1047–1057. doi: 10.1128/JB.183.3.1047-1057.2001 11208804 PMC94973

[B31] Ohman D. E. Chakrabarty A. M. (1981). Genetic mapping of chromosomal determinants for the production of the exopolysaccharide alginate in a *Pseudomonas aeruginosa* cystic fibrosis isolate. Infect. Immun. 33, 142–148. doi: 10.1128/iai.33.1.142-148.1981 6790439 PMC350668

[B32] Pier G. B. Coleman F. Grout M. Franklin M. Ohman D. E. (2001). Role of alginate O acetylation in resistance of mucoid *Pseudomonas aeruginosa* to opsonic phagocytosis. Infect. Immun. 69, 1895–1901. doi: 10.1128/IAI.69.3.1895-1901.2001 11179370 PMC98099

[B33] Pils S. Schmitter T. Neske F. Hauck C. R. (2006). Quantification of bacterial invasion into adherent cells by flow cytometry. J. Microbiol. Methods 65, 301–310. doi: 10.1016/j.mimet.2005.08.013 16185780

[B34] Rubin B. K. (2007). Mucus structure and properties in cystic fibrosis. Paediatric Respir. Rev. 8, 4–7. doi: 10.1016/j.prrv.2007.02.004 17419972

[B35] Ruhen R. W. Holt P. G. Papadimitriou J. M. (1980). Antiphagocytic effect of pseudomonas aeruginosa exopolysaccharide. J. Clin. Pathol. 33, 1221–1222. doi: 10.1136/jcp.33.12.1221 6778898 PMC1146384

[B36] Sadikot R. T. Blackwell T. S. Christman J. W. Prince A. S. (2005). Pathogen-host interactions in pseudomonas aeruginosa pneumonia. Am. J. Respir. Crit. Care Med. 171, 1209–1223. doi: 10.1164/rccm.200408-1044SO 15695491 PMC2718459

[B37] Schepetkin I. A. Quinn M. T. (2006). Botanical polysaccharides: macrophage immunomodulation and therapeutic potential. Int. Immunopharmacol. 6, 317–333. doi: 10.1016/j.intimp.2005.10.005 16428067

[B38] Sun X. Wang X. Chen T. Li T. Cao K. Lu A.. (2010). Myelin activates FAK/Akt/NF-kappaB pathways and provokes CR3-dependent inflammatory response in murine system. PloS One 5, e9380. doi: 10.1371/journal.pone.0009380 20186338 PMC2826415

[B39] Tabary O. Zahm J. M. Hinnrasky J. Couetil J. P. Cornillet P. Guenounou M.. (1998). Selective up-regulation of chemokine IL-8 expression in cystic fibrosis bronchial gland cells *in vivo* and *in vitro* . Am. J. Pathol. 153, 921–930. doi: 10.1016/S0002-9440(10)65633-7 9736040 PMC1853001

[B40] Tart A. H. Blanks M. J. Wozniak D. J. (2006). The AlgT-dependent transcriptional regulator AmrZ (AlgZ) inhibits flagellum biosynthesis in mucoid, nonmotile pseudomonas aeruginosa cystic fibrosis isolates. J. Bacteriol 188, 6483–6489. doi: 10.1128/JB.00636-06 16952938 PMC1595476

[B41] Tart A. H. Wolfgang M. C. Wozniak D. J. (2005). The alternative sigma factor AlgT represses pseudomonas aeruginosa flagellum biosynthesis by inhibiting expression of fleQ. J. Bacteriol 187, 7955–7962. doi: 10.1128/JB.187.23.7955-7962.2005 16291668 PMC1291279

[B42] Thieblemont N. Haeffner-Cavaillon N. Haeffner A. Cholley B. Weiss L. Kazatchkine M. D. (1995). Triggering of complement receptors CR1 (CD35) and CR3 (CD11b/CD18) induces nuclear translocation of NF-kappa b (p50/p65) in human monocytes and enhances viral replication in HIV-infected monocytic cells. J. Immunol. 155, 4861–4867. doi: 10.4049/jimmunol.155.10.4861 7594489

[B43] Tsuchiya S. Kobayashi Y. Goto Y. Okumura H. Nakae S. Konno T.. (1982). Induction of maturation in cultured human monocytic leukemia cells by a phorbol diester. Cancer Res. 42, 1530–1536. 6949641

[B44] Underhill D. M. Goodridge H. S. (2012). Information processing during phagocytosis. Nat. Rev. Immunol. 12, 492–502. doi: 10.1038/nri3244 22699831 PMC5570470

[B45] Wood L. F. Leech A. J. Ohman D. E. (2006). Cell wall-inhibitory antibiotics activate the alginate biosynthesis operon in *Pseudomonas aeruginosa*: roles of sigma (AlgT) and the AlgW and prc proteases. Mol. Microbiol. 62, 412–426. doi: 10.1111/j.1365-2958.2006.05390.x 17020580

[B46] Wood L. F. Ohman D. E. (2009). Use of cell wall stress to characterize sigma 22 (AlgT/U) activation by regulated proteolysis and its regulon in pseudomonas aeruginosa. Mol. Microbiol. 72, 183–201. doi: 10.1111/j.1365-2958.2009.06635.x 19226327

[B47] Yang D. Jones K. S. (2009). Effect of alginate on innate immune activation of macrophages. J. Biomed. materials Res. Part A 90, 411–418. doi: 10.1002/jbm.a.32096 18523947

[B48] Zhou H. Liao J. Aloor J. Nie H. Wilson B. C. Fessler M. B.. (2013). CD11b/CD18 (Mac-1) is a novel surface receptor for extracellular double-stranded RNA to mediate cellular inflammatory responses. J. Immunol. 190, 115–125. doi: 10.4049/jimmunol.1202136 23209319 PMC3529770

