# Supplementary figures and images for: Mechanism of resistance to phagocytosis and pulmonary persistence in mucoid Pseudomonas aeruginosa
Source: Front Cell Infect Microbiol. 2023 Mar 15;13:1125901. doi: 10.3389/fcimb.2023.1125901 (PMC10050686; doi:10.3389/fcimb.2023.1125901)

**Supplemental Material**

**Rowe et al.**

Fig. S1

A.


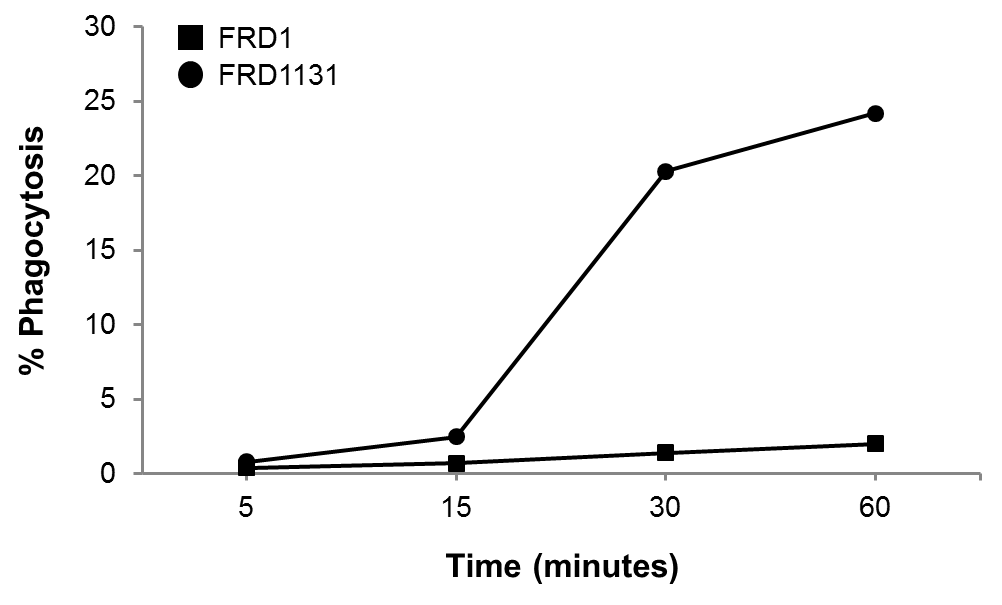


B.


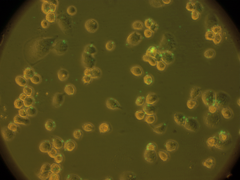

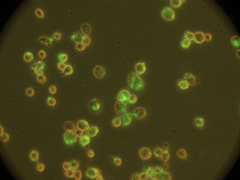


FRD1 (Alg+ GFP+) FRD1131 (Alg- GFP+)

Fig. S2


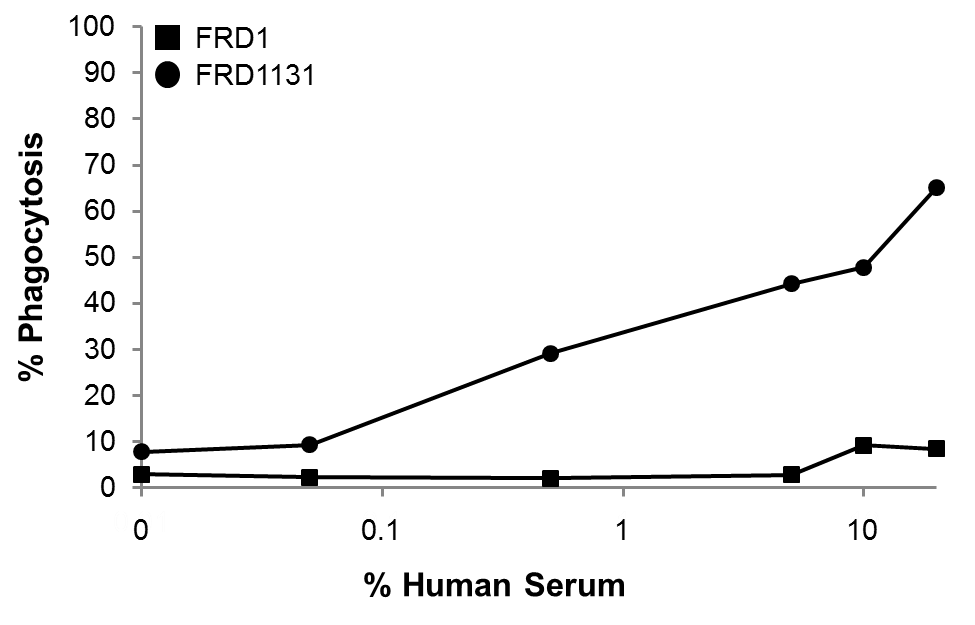


Fig. S3


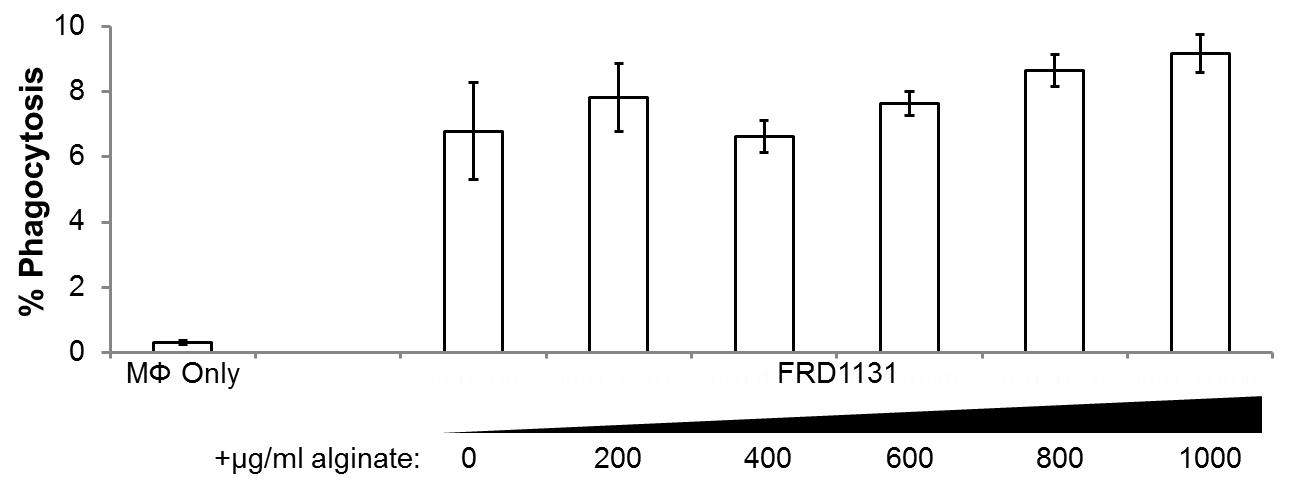


Fig. S4


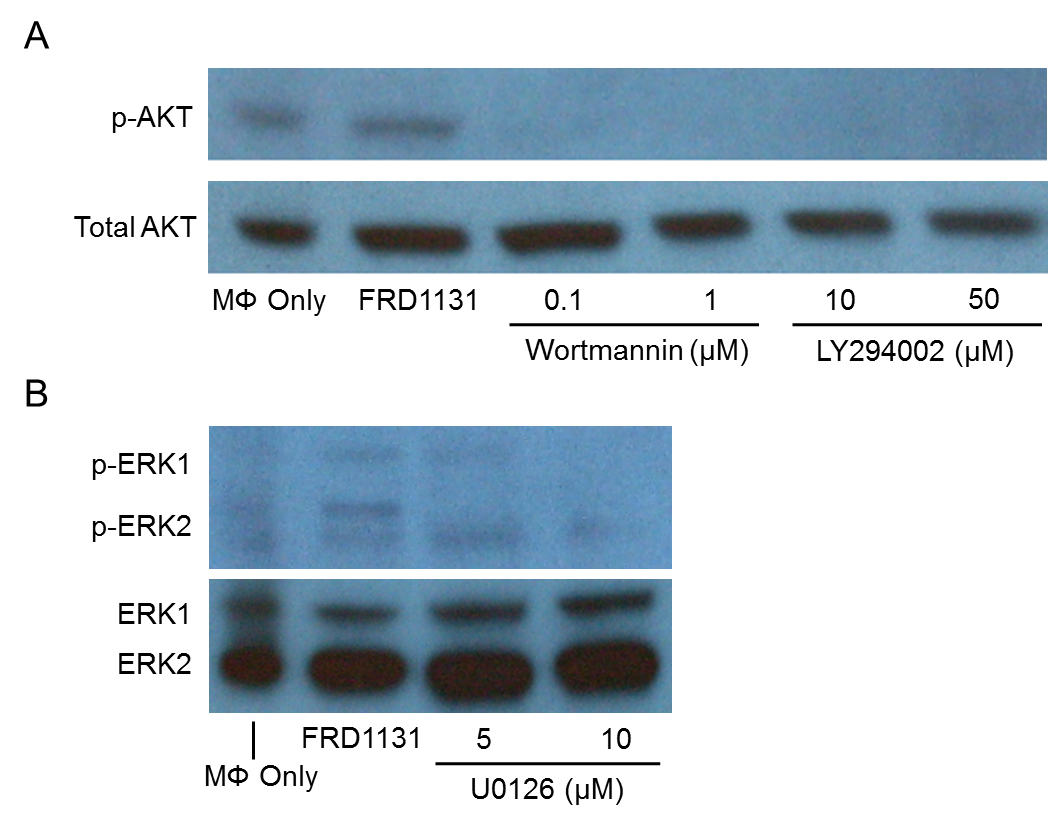

Supplement: Supplementary Figure 1 — Time course of phagocytosis of FRD1 and FRD1131 by MH-S macrophages. (A) MH-S macrophages were infected with GFP-expressing Alg+ FRD1 or Alg- FRD1131 for various time points up to 1 h. Percent phagocytosis indicates the percent of macrophages that are associated with GFP-expressing P. aeruginosa as detected by flow cytometry. Reduced phagocytosis of FRD1 compared to FRD1131 was observed as early as 30 min. Data represent one typical experiment of several performed. (B) Visualization of THP-1 macrophages following phagocytosis of GFP-expressing FRD1 and FRD1131. Images show GFP-expressing P. aeruginosa overlaid on a visible light image of THP-1 macrophages. THP-1 macrophages were infected with GFP-expressing FRD1 or FRD1131 for 30 min and visualized by fluorescent microscopy. Inhibited phagocytosis, and association, was observed with FRD1 compared to FRD1131. [file DataSheet_1.doc]
